# The Neuromuscular Junction Distribution in the Upper Face: An Anatomy‐to‐Practice Review to Inform Botulinum Toxin Type A Treatment Planning

**DOI:** 10.1111/jocd.70921

**Published:** 2026-05-13

**Authors:** Fabiano Nadson Magacho‐Vieira

**Affiliations:** ^1^ Magacho Institute for Health Education Fortaleza Ceará Brazil; ^2^ Department of Clinical, Aesthetic, and Surgical Dermatology Batista Memorial Hospital Fortaleza Ceará Brazil

**Keywords:** botulinum toxin, corrugator supercilii, depressor supercilii, facial anatomy, frontalis, neuromuscular junction, orbicularis oculi, procerus

## Abstract

**Background:**

Upper‐face botulinum toxin treatment planning often relies on surface landmarks and standardized injection patterns, yet neuromuscular junctions are distributed in muscle‐specific motor zones that vary in topography, depth, and overlap. Injecting near an optimal motor zone has been associated with greater clinical effect, suggesting that mapping these patterns may refine anatomy‐guided targeting and reduce variability.

**Aims:**

To synthesize anatomical evidence on micro‐innervation and neuromuscular junction clustering in the frontalis, corrugator supercilii, orbicularis oculi, procerus, and depressor supercilii.

**Methods:**

A clinician‐oriented “anatomy‐to‐practice” narrative review was conducted. Studies with extractable human, muscle‐level micro‐innervation mapping were synthesized qualitatively, emphasizing cluster location, depth, and distribution.

**Results:**

Neuromuscular junctions were nonuniformly distributed, forming muscle‐specific distribution patterns, ranging from more discrete, clustered territories to diffuse fields. They tended to concentrate in the mid‐ to upper‐frontalis belly along the deep fascial surface; in the corrugator supercilii, predominantly within the medial portion and at greater depth, centrally and inferiorly along the orbital rim in the depressor supercilii; and inferiorly near the nasion in the procerus. In the orbicularis oculi, neuromuscular junctions were diffusely distributed across the muscle. Given heterogeneous and often proxy‐based mapping, these territories are best interpreted as probabilistic fields.

**Conclusions:**

Micro‐innervation and neuromuscular junction distribution represent a complementary anatomical layer that may inform upper‐face botulinum toxin treatment planning alongside dynamic assessment, clinical judgment, and established safety considerations.

## 1 Introduction

Upper‐face mimetic muscles are primary targets for botulinum neurotoxin type A (BoNT‐A) in the treatment and prevention of dynamic rhytides [1, 2]. BoNT‐A exerts its effect at the presynaptic terminal of the neuromuscular junction (NMJ), and clinical outcomes are partially dependent on how closely injections are placed to endplate‐rich territories where NMJs cluster, so‐called motor zones (MZs) [1, 3, 4]. Consistent with this, injecting as little as 1 cm from an optimal MZ has been associated with up to a 46% reduction in clinical effect [5].

Unlike limb muscles, facial mimetic muscles have small fibers, limited fascial compartmentalization, and frequent interdigitation, favoring diffuse or multifocal NMJ clustering [6, 7, 8, 9]. Mapping muscle‐specific MZ locations may facilitate and accelerate neurotoxin binding at the presynaptic terminal and, consequently, may reduce toxin wastage, support a faster onset of clinical response, and reduce the risk of aberrant spread and unnecessary inhibition of neighboring muscles [3, 5, 6, 9, 10].

This review synthesizes anatomical evidence on motor micro‐innervation and NMJ cluster topography in the frontalis, corrugator supercilii (CSM), orbicularis oculi (OOc), procerus, and depressor supercilii (DSM) muscles to inform anatomy‐guided BoNT‐A planning.

## 2 Methods

A structured narrative anatomical review was conducted. This format was selected because the available evidence was methodologically heterogeneous and not suitable for formal quantitative pooling. PubMed/MEDLINE was searched from inception to February 27, 2026, combining muscle‐specific terms (*frontalis*, *corrugator supercilii*, *orbicularis oculi*, *procerus*, and *depressor supercilii*) with NMJ/innervation concepts and BoNT‐A‐related terms; backward and forward citation tracking were used to complement database retrieval. No language restrictions were applied, although preference was given to English‐language articles. Records were screened by a single reviewer at the title/abstract and full‐text levels, with priority given to human studies providing extractable, muscle‐level mapping data relevant to injection planning. Evidence was synthesized qualitatively by muscle, with emphasis on NMJ cluster location, depth, and distribution patterns. The complete search syntax and screening procedure are provided in the Supporting Information.

## 3 Results and Anatomical Review

Upper facial musculature is governed by the motor nucleus of the facial nerve (cranial nerve VII) through bilateral central cortical innervation via the corticobulbar tract, thus receiving commands from both cerebral hemispheres [11, 12]. The specific innervation patterns for each individual muscle diverge, as will be detailed in the following sections. A consolidated, cross‐muscle overview of macro‐innervation, intramuscular arborization, and MZ probable locations is provided in Table 1.

**TABLE 1:** Macro‐innervation, intramuscular micro‐innervation, and neuromuscular junction cluster distribution in upper‐face muscles.

Muscle (subdivision)	Macro‐innervation	Intramuscular micro‐innervation (arborization)	NMJ cluster (motor endplate)
Frontalis	Frontal (temporal) branch; approaches laterally and supplies the muscle from its deep surface; terminal branches predominantly enter the undersurface of the inferior and middle thirds [8, 13, 14]	Deep‐plane branches form an intramuscular plexus and arborize extensively within the muscle fiber bundles along the deep fascial plane [8, 15]	*Topography*: Distal endings/endplates concentrate in central transverse territories (middle‐to‐upper belly), with relative sparsity near the hairline and inferiorly near the orbital rim; band‐shaped clusters may appear continuous or separated into medial and lateral clusters; richer arborization reported ~2.5–3.0 cm above the superior orbital margin or 1.5–2.0 cm above the eyebrow [8, 15, 16] *Depth*: NMJ clusters described as closely associated with the deep fascial bed, consistent with deep‐surface motor entry [8, 14]
Corrugator supercilii—transverse head	Transverse/superior portion supplied by the frontal (temporal) branch, penetrating the deep surface in the supraorbital region [17, 18]	Temporal branch attenuates into a fine plexus that penetrates the deep surface of the transverse head [17].	*Topography*: Multifocal entry‐territory clustering near temporal‐branch penetration; reported penetration ~7.6 mm above the superior orbital rim and ~6.3 mm lateral to the supraorbital foramen [19] *Depth*: Depth is course‐dependent with a medial‐to‐lateral gradient: deeper medially (reported 4.71–5.50 mm skin‐to‐muscle; mean up to 7.5 ± 1.6 mm in the osseous anchoring zone) and more superficial laterally (~4.64–5.11 mm at the mid‐pupillary line) [20, 21]
Corrugator supercilii—oblique head	Inferomedial portion is supplied by the angular nerve; the supraorbital/supratrochlear sensory nerves may traverse the muscle without motor function [18, 22, 23]	Angular nerve preferentially supplies the inferomedial oblique head; terminal motor branches concentrate neuromuscular contacts near entry points; deep sensory supraorbital branches may course along the deep surface parallel to muscle fibers and pierce toward the skin [18, 22]	*Topography*: Cluster weighting shifts inferomedially toward angular/zygomatic‐supplied territories; reported courses are ~4–7 mm medial to the medial canthus; maximal functional responsiveness may lie slightly more laterally along the frown vector (mid‐brow) [18, 24, 25] *Depth*: Same course‐dependent medial‐to‐lateral depth gradient, with deeper medial targets and progressive superficialization superolaterally [20, 21]
Orbicularis oculi—orbital part	Dense motor plexus primarily from temporal and zygomatic branches (with variable additional buccal rami) approaches from the posterior surface and enters near peripheral attachments along the orbital rim [1, 10, 17]	Intercommunicating deep‐surface plexus with diffuse, multifocal, mesh‐like arborization across the orbital region [1, 9, 26]	*Topography*: Diffuse “field” clustering. Based on neural mapping (Sihler's stain), higher‐density territories of terminal nerve branches (inferring NMJ density) are reported superiorly in the superolateral arc (9–12 o'clock) and inferiorly in inferomedial‐to‐inferolateral arcs (clockface mapping) [1, 10] *Depth*: Extremely flat and superficial beneath skin/superficial fascia; mean epidermis‐to‐muscle surface depth of ~4.7–5.5 mm is reported in lateral periorbital ultrasound, with posterior/deep‐surface neural entry [1, 6]
Orbicularis oculi—preseptal part	Supplied within the same facial‐nerve plexus (temporal/zygomatic‐dominant) entering from the posterior surface [1, 10]	Diffuse arborization with fine terminal twigs distributed through preseptal fibers as part of the intercommunicating plexus [9, 26]	*Topography*: Less uniform distribution, with greater representation in medial and lateral thirds and significantly lower frequency centrally [27] *Depth*: Superficial muscle plane with posterior/deep‐surface neural entry [1]
Orbicularis oculi—pretarsal part	Supplied within the same facial‐nerve plexus distributed across pretarsal fibers [1, 10]	Fine twigs consistent with short, serially arranged fibers and staggered endplate organization [9, 27]	*Topography*: Small NMJ clusters in a staggered pattern along the full mediolateral span; described as relatively high‐density (twofold greater neuromuscular junction density compared to the preseptal region) [27, 28] *Depth*: Superficial plane with posterior/deep‐surface entry [1]
Procerus	Primary motor supply from the angular nerve; terminal fibers course superiorly along the medial canthus and enter the muscle predominantly through its lateral (temporal) margin and posterior (deep) surface [18, 24, 26]	Small terminal branches are distributed along the deep (posterior) interface of the muscle, and muscle fibers propagate in a cranial and cranio‐lateral direction [26, 29]	*Topography*: Motor endplates form clusters in the immediate vicinity of nerve entrance points, being primarily concentrated in the inferior (caudal) portion of the muscle belly near its origin at the nasal root [9, 29] *Depth*: Thin belly (≤ 1 mm), described as ~2–3 mm beneath the cutaneous surface; MEZs are therefore constrained to this superficial depth at the posterior interface [2, 18]
Depressor supercilii	Innervated by medial facial‐nerve branches (derived from superficial zygomatic, bucco‐zygomatic, or buccal branches) that pass anteriorly to the medial palpebral ligament. The muscle originates securely from the frontal process of the maxilla [24, 30]	Terminal nerve branches penetrate and ramify inside the muscle near its inferior osseous attachment [24]	*Topography*: NMJ clusters are inferred as densest near the inferior osseous origin and distributed across the short (~4–5 mm) vertical subcutaneous span [2, 30] *Depth*: Superficial mimetic plane directly beneath the skin, passing over and distinct from the deeper medial corrugator belly [30, 31]

*Note:* Summary of the available anatomical evidence for the frontalis, corrugator supercilii, orbicularis oculi, procerus, and depressor supercilii muscles, including patterns of motor nerve supply, intramuscular arborization, and the topography and approximate depth of neuromuscular junction‐rich territories.

### 3.1 Frontalis: Transverse Band‐Like MZ

The frontalis muscle is innervated exclusively by the frontal (temporal) branch of the facial nerve [13]. Macroscopic dissections demonstrate that these branches approach laterally, coursing deep to the frontalis to supply it from its posterior aspect, where they ascend and arborize in a superomedial direction along the deep fascial plane [8, 13, 14].

Distal nerve endings, acting as topographic proxies for MZs, are distributed with consistently higher density toward the middle and upper thirds of the frontalis belly, leaving the superior and inferior peripheral regions less populated [15]. High‐density surface electromyography provides direct in vivo corroboration of this macro‐architecture, demonstrating that functional NMJs are predominantly localized in the upper half of the forehead and may organize either as a continuous transverse band or as two separate (medial and lateral) clusters [16]. This functional mapping also revealed that action potentials propagate bidirectionally from these endplates; while superior medial fibers run in a straight cranial direction, the propagation vector becomes increasingly cranio‐lateral in more laterally located motor units, demonstrating a functional fan‐like divergence at the muscle's periphery [16]. Direct histological and immunohistochemical visualization confirmed these functional and topographic inferences and added that NMJs aggregate centrally within the muscle belly and are closely tethered to its deep fascial aspect [8]. It also showed an unexpectedly dense population of atypical, voluminous nerve endings interfacing with the muscle fibers at the superior terminal transition zone, where the frontalis fibers coalesce with the galea aponeurotica [8].

Complementing this macroscopic perspective, Yi et al. [17] recently proposed surface‐anchored injection points for horizontal forehead lines based on external landmarks of the forehead and on regional variation in frontalis thickness, providing additional cadaveric reference data for clinical localization.

### 3.2 CSM: Deep Medial MZ

The CSM muscle is innervated by the temporal branch of the facial nerve in 66% of cases, the zygomatic branch in 17%, and the angular nerve in 17% [18]. These branches enter the muscle via one (29.3%) or two (70.7%) rami [19]. Topographical measurements demonstrate that these branches insert into the CSM at a point averaging 7.6 ± 2.6 mm higher than the superior orbital margin and 6.3 ± 3.5 mm lateral to the supraorbital foramen [19].

Although terminal rami enter the lateral CSM, high‐density surface electromyography suggests this lateral third is largely devoid of NMJs [16]. As the nerve courses medially, endplates start to appear in the middle third and concentrate into a functional cluster in the medial third [16]. Histological evaluations using muscle biopsies confirm the presence of those microscopic NMJ clusters within the CSM muscle [20]. Crossing macroscopic and three‐dimensional mapping data [21, 22], it is possible to propose a consolidated topographic and depth framework for the CSM MZ, locating it at a medial origin approximately 2.9 mm from the nasion, consistently at the intersection of the vertical line through the medial canthus and the horizontal line through the glabella, and at a comparable depth of 5.4–6.6 mm from the skin surface.

### 3.3 OOc: Superficial, Diffusely Staggered NMJs

The OOc muscle is innervated by the temporal, zygomatic, and buccal branches of the facial nerve [10, 23]. Macroscopic dissections demonstrate that these branches assemble on the posterior surface of the muscle to form a mesh‐like plexus [23]. The muscle micro‐architecture consists of short myofibers arranged in series that do not extend the full length of the eyelid [24], with a reported presence of multifocally innervated muscle fibers and individual facial fibers bearing two to five NMJs [9].

Çiçek et al. [10] used a clock model (12 o'clock superior, 3 o'clock medial) and reported higher nerve‐branch density superolaterally above the palpebral fissure and below it (right: 4–7 o'clock; left: 5–8 o'clock). Wirtschafter et al. [24] also mapped those regional differences for NMJ distribution: Pretarsal NMJs are diffusely scattered across the entire eyelid, whereas preseptal NMJs concentrate medially and laterally, remaining sparse centrally.

Direct histological visualization shows NMJs are spread evenly over the muscle, appearing either isolated or grouped into a great number of small MZs [1, 9]. This diffuse distribution is corroborated in vivo by high‐density surface electromyography, demonstrating that endplates are distributed over the entire muscle with high interindividual variability, frequently forming clusters lateral to the outer canthus, in the latero‐cranial part, and cranial and caudal to the pupil [16, 25].

### 3.4 DSM: NMJs Cluster in the Central–Inferior Region

The DSM muscle is an anatomically and histologically distinct muscle from the CSM and the medial head of the OOc [26]. It originates from the frontal process of the maxilla approximately 10 mm above the medial canthal tendon, frequently arising from two distinct heads, and courses directly upward to insert into the dermis approximately 14–15 mm superior to the medial canthal tendon [2, 26].

The motor innervation for the DSM is supplied by the zygomatic branch of the facial nerve [16]. In vivo high‐density surface electromyography has successfully mapped DSM NMJs, showing a clustered distribution in the central–caudal portion of the muscle, at the lateral glabella, along the orbital rim region, superior to the medial canthus [16].

### 3.5 Procerus: Inferior MZ, Near the Muscle Origin

The procerus is a superficial, pyramidal‐shaped muscle that originates over the lower nasal bone and upper nasal cartilage, inserting into the skin between the eyebrows and into the frontalis muscle [2, 27]. Topographical measurements indicate that it is a very thin muscle, less than 1 mm thick, and its muscular belly is located at an average depth of 2–3 mm beneath the surface of the skin [2].

Macroscopic dissections demonstrate distinct anatomical pathways regarding the motor innervation of the procerus muscle, typically penetrating the muscle at its temporal (lateral) margin and its posterior surface [16, 23, 28, 29]. High‐density surface electromyography demonstrated that NMJs are primarily found as clusters in the inferior portion of the muscle near its origin. Additional endplates are found loosely distributed in the mediolateral direction and are rarely determined in a more superior position [16].

## 4 Discussion

BoNT‐A diffusion appears inversely related to local presynaptic receptor density [30]. NMJ‐rich territories (heat zones) may function as a biological sponge, rapidly adsorbing and sequestering active molecules, limiting passive dispersion to adjacent tissues [30]. By contrast, NMJ‐sparse “cold zones” present fewer available binding sites, which may permit greater diffusion into unintended neighboring musculature [6, 27]. Compensatory dose escalation in receptor‐poor areas may further increase the risk of aberrant spread and adverse effects (e.g., brow or eyelid ptosis, asymmetry). Current anatomical understanding of NMJ distribution may help refine botulinum toxin treatment planning.

### 4.1 Why Maps Diverge and How to Interpret Them in Clinical Practice

**FIGURE 1:**
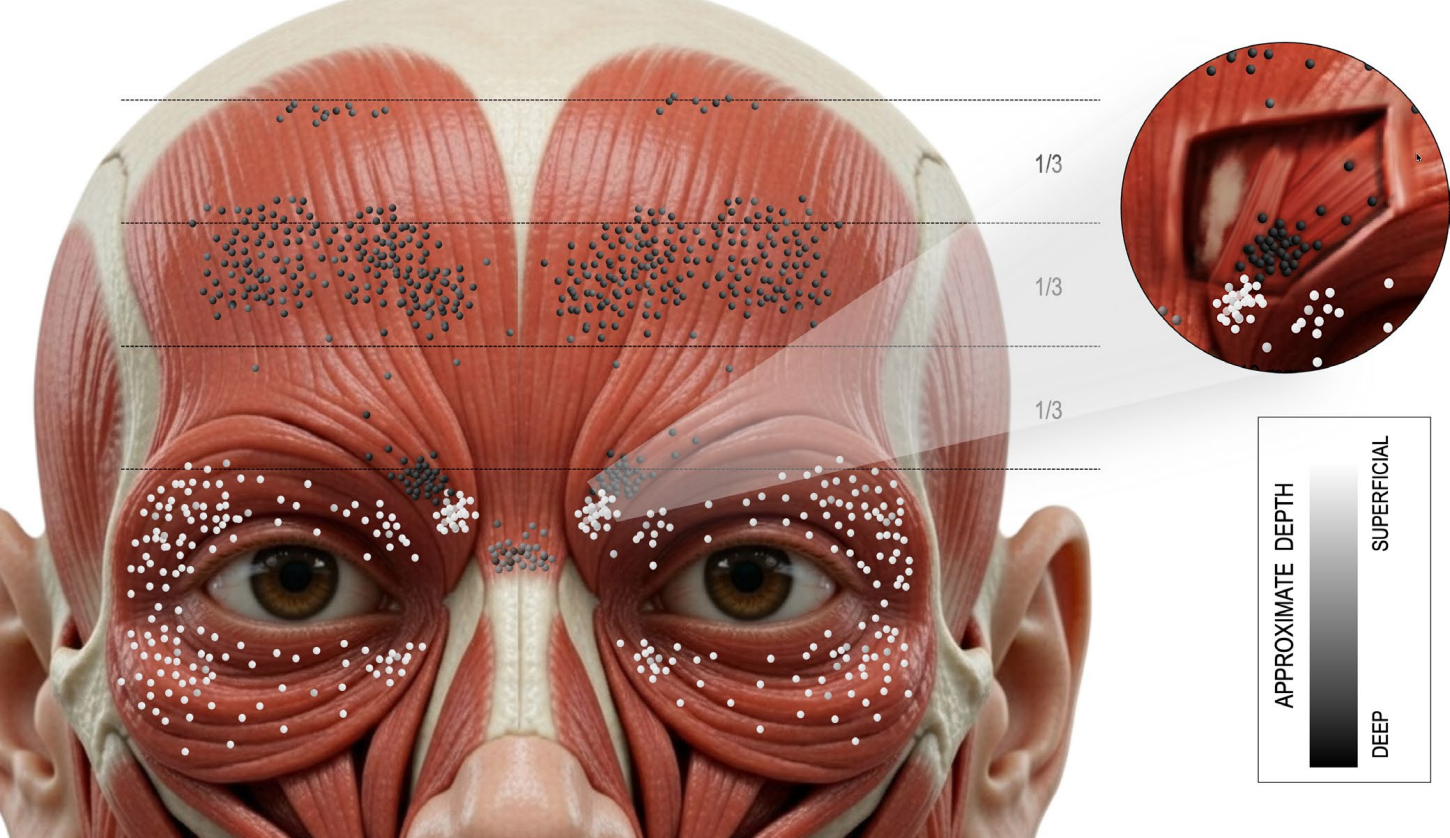
Depth‐aware probabilistic map of upper‐face motor zones relevant to botulinum toxin type A treatment planning. Schematic summary of the most probable neuromuscular junction‐rich territories in the frontalis, corrugator supercilii, orbicularis oculi, depressor supercilii, and procerus muscles, integrating topography, approximate depth, and regional overlap. Grayscale indicates approximate depth from superficial to deep. The inset illustrates the layered relationship and partial overlap between the deep medial corrugator supercilii and the more superficial depressor supercilii. This figure should be interpreted as an anatomical reference rather than as a prescriptive injection template.

Clinically, a pragmatic approach is to interpret these “hot zones” as depth‐aware probabilistic fields defined by topography, depth, and overlap, rather than as fixed surface marks or a single “motor point.” Within this framework, the present synthesis proposes an evidence‐weighted, depth‐aware probabilistic map of upper‐face MZs (Figure 1), intended as an anatomical reference to contextualize targeting rather than as a treatment directive. In integrating the available data, evidence was prioritized in the following order: direct human histology and immunohistochemistry, followed by in vivo high‐density surface electromyography, macroscopic intramuscular nerve arborization, and cadaveric topographic measurements used to anchor surface coordinates and approximate depth. No formal consensus method was applied; Figure 1 represents a qualitative integration of the evidence summarized in Table S1.

### 4.2 Distribution of NMJs and Injection Depth in the Frontalis Muscle

As previously noted, distal nerve endings are comparatively sparse at the superior and inferior margins of the frontalis muscle, implying that injections in these regions, particularly near the superior midline, may result in inefficient receptor engagement [15]. In contrast, the inferior frontalis remains clinically sensitive: When neuromodulation is pursued near the brow for shaping purposes, conservative, intent‐driven placement is typically recommended to reduce the likelihood of unintended brow ptosis [2, 27].

Additionally, fibers of the temporal branch of the facial‐nerve approach the frontalis from its posterior aspect [13, 14], and most NMJs appear to be anchored to the deep fascial surface of the muscle belly [8]. Taken together, these observations support the rationale for placing BoNT‐A in deeper layers when the goal is to target NMJ‐rich zones. Nevertheless, superficial placement remains common in clinical practice, and satisfactory outcomes have been reported [31, 32]. One potential explanation relates to regional anatomy. The frontalis is a thin muscle (500–900 μm) [8], with a delicate superficial epimysium that is closely connected to the dermis by fibrous septa [33]. The anterior surface of the frontalis lies at a mean depth of only 2.82 mm in females and 2.97 mm in males [34]. This microanatomy may facilitate diffusion from superficial injections toward the muscle fibers [31].

Davidovic et al. [35] reported greater motor relaxation during maximal contraction when BoNT‐A was placed in a deep supraperiosteal plane versus subcutaneous injection. On a cautionary note, Welter et al. [8] advise that deep injections traversing the deep fascia would, in contrast, limit the distribution of the injected solution toward the muscle fibers, increasing spread.

While the optimal depth remains in debate, current evidence appears to suggest that targeting the deeper muscle belly may be associated with more robust frontalis relaxation. Superficial injections may also be effective, while limiting equimosis and eyebrow heaviness and ptosis [31, 32]. No high‐level comparative clinical evidence currently favors one injection plane over another for the frontalis. Ultimately, the preferred injection plane may indeed depend on the desired clinical endpoint.

### 4.3 Glabellar Complex: Safety First and Depth Congruence

The three‐dimensional coordinates for the CSM MZ presented in this review integrate evidence from distinct topographical approaches [21, 22] and show close agreement with independent anatomical assessments, including the systematic review by Hwang et al. [18]. Their synthesis indicates that the depth of the CSM surface remains relatively constant as the muscle courses laterally. This structural consistency challenges the common clinical assumption—sometimes reiterated in recent literature—that lateral injections must be placed subdermally or intradermally in order to target a presumed dermal insertion of the muscle.

Foundational anatomical guidance, including Yi et al. [36], suggests that the rationale is primarily safety‐driven. Proximate to the mid‐pupillary region, the deep fascia of the corrugator lies near neurovascular bundles that traverse the orbital septum. Exploratory injection and dissection studies further suggest that the fascial sheaths surrounding these bundles can act as conduits, facilitating retrograde spread through the orbital septum into the intraorbital space, with potential involvement of the levator palpebrae superioris [37, 38]. Accordingly, the traditional recommendation to inject superficially in the lateral corrugator remains well justified, not because the muscle is confined to the dermis but as a pragmatic safety measure to reduce exposure to deeper neurovascular routes and mitigate the risk of inadvertent blepharoptosis.

Because the periorbital region is so functionally unforgiving, diffusion concerns reinforce why depth awareness remains central when translating anatomy to practice [6, 27, 37]. In the medial CSM region, this is further supported by the direct topographic overlap: the DSM courses superficially over the deep origin of the corrugator, partially covering its oblique head [26]. Extrapolating that anatomical and functional localization data, when the intended target is the DSM (rather than the CSM), a superficial, subdermal/intradermal injection may better align with the central–inferior clustering of its endplates along the orbital rim, near its osseous origin, approximately 10 mm superior to the medial canthus [16, 26]. However, this recommendation should be interpreted as anatomy‐informed guidance rather than a clinically validated technique; to the author's knowledge, no dedicated clinical outcome trial of isolated DSM targeting has been published.

### 4.4 Injection Points for Crow's Feet Treatment With BoNT‐A

Because the NMJs in the OOc muscle are diffusely scattered, it has been hypothesized that modifying injection strategies, such as using a higher number of injection points, employing retrograde techniques, or adjusting reconstitution volumes, may optimize neurotoxin uptake across the targeted area [1, 39, 40]. Despite this anatomical rationale, clinical trials [39, 41, 42, 43] have yielded mixed results.

This divergence in clinical outcomes may be partly explained by methodological factors. However, the previously established absence of significant MZs, together with the nonhomogeneous distribution of crow's feet patterns [44], suggests that while standardized “on‐label” templates serve as foundational guides, optimal outcomes likely rely on highly individualized approaches [20].

In this context, splitting the total dosage into numerous microdroplets dispersed over the treatment area offers a strategy conceptually aligned with the diffuse NMJ pattern of the OOc [45]. This approach was clinically introduced for crow's feet by Imhof and Kuhne [46] in a split‐face design, citing the diffuse innervation of the OOc as its anatomical rationale, and was subsequently supported by Cao et al. [47] in a comparative in vivo study showing efficacy of intradermal microdroplets for both dynamic and static lateral canthal lines. Microdroplet placement has also been described as particularly suited to the delicate lower portion of the OOc. Mechanistically, this strategy may plausibly reduce focal saturation and limit diffusion into adjacent mid‐facial musculature, which is particularly relevant in a thin, functionally unforgiving neighborhood [6]. However, direct clinical validation specifically targeting the NMJ topography of the OOc is limited.

### 4.5 Neuroplasticity After Repeated Treatments: Potential Implications for Dosing Paradigms

Human NMJs themselves remain remarkably stable without signs of morphological degeneration across the entire adult lifespan [48], but three‐dimensional mapping supports spatial adaptation of endplate distributions following denervation [49]. In parallel, long‐term BoNT‐A exposure is also consistent with remodeling and/or reinnervation processes [50]. Histologic studies of repeatedly treated muscles describe presynaptic remodeling, including axonal sprouting and de novo NMJ formation, alongside polyneuronal innervation patterns and altered features of synapse elimination [51, 52, 53]. In some contexts, repeated exposure has also been associated with increased nerve fiber density, augmented NMJ numbers, and myofiber changes [53]. Collectively, these dynamics may offer a biologically plausible framework for why dose adjustment or escalation would sometimes be considered across treatment cycles. However, because much of the supporting evidence derives from therapeutic contexts, the extent to which these processes apply to typical esthetic regimens remains uncertain and presents an avenue for future investigation.

### 4.6 Limitations

Several included studies were performed in non‐esthetic contexts, and anatomical landmarks may vary across individuals. Evidence is limited and sometimes conflicting, and many “mapping” approaches rely on indirect proxies rather than direct NMJ visualization. Some proposed localizations are therefore inferential. As records were screened by a single reviewer, selection bias cannot be excluded.

## 5 Conclusion

Overall, this synthesis is intended to draw attention to micro‐innervation and MZ topography and depth as a complementary microanatomical layer that may be considered alongside established macroscopic anatomy and dynamic assessment in upper‐face BoNT‐A planning. It is designed not to replace but to complement dynamic assessment, clinician judgment, and established safety considerations.

## Author Contributions

**Fabiano Nadson Magacho‐Vieira:** conceptualization, writing – original draft, writing – review and editing. The author reviewed, approved, and agreed to be accountable for all aspects of the final version of this article.

## Funding

The author has nothing to report.

## Ethics Statement

The author has nothing to report.

## Consent

The author has nothing to report.

## Conflicts of Interest

The author declares no conflicts of interest.

## Supporting information


**Data S1:** Supplementary methods.

**Table S1:** Anatomical evidence underlying the depth‐aware probabilistic map of upper‐face motor zones (Figure 1).

## Data Availability

This is a narrative review article with no primary research data. The full search syntax, screening procedure, and source‐by‐source evidence summary underlying the synthesis are provided in the Supporting Information of this article.
