# Supplementary material for: The Neuromuscular Junction Distribution in the Upper Face: An Anatomy‐to‐Practice Review to Inform Botulinum Toxin Type A Treatment Planning
Source: J Cosmet Dermatol. 2026 May 13;25:e70921. doi: 10.1111/jocd.70921 (PMC13172661; doi:10.1111/jocd.70921)
Supplement: Supplementary file 1 — Data S1: Supplementary methods. [file JOCD-25-e70921-s001.docx]

**Supplementary Methods**

*The neuromuscular junction distribution in the upper face: An anatomy-to-practice review to inform botulinum toxin type A treatment planning*

Manuscript ID: 2301778 — Journal of Cosmetic Dermatology

**S1. Review framing and rationale**

A structured narrative anatomical review was conducted to synthesize the evidence on motor microinnervation and neuromuscular junction (NMJ) cluster topography in the upper-face musculature. The narrative format was preferred over a systematic review because the available literature is methodologically heterogeneous — encompassing cadaveric histology, immunohistochemistry, modified Sihler staining, three-dimensional cadaveric mapping, and in vivo high-density surface electromyography (HD-sEMG) — and reports outcomes that are not amenable to formal quantitative pooling.

**S2. Database, period, and search syntax**

PubMed/MEDLINE was searched from inception to 27 February 2026. Predefined Boolean combinations were applied, pairing muscle terms with NMJ/motor endplate and localization concepts and with botulinum toxin type A–related terms to capture injection-planning reports. The full set of terms used is summarized below.

**Muscle terms:**

frontalis OR “corrugator supercilii” OR “orbicularis oculi” OR procerus OR “depressor supercilii”

**NMJ / innervation / localization concepts:**

“neuromuscular junction” OR “motor endplate” OR “innervation zone” OR “motor point” OR “intramuscular innervation” OR microinnervation OR “endplate distribution”

**BoNT-A / injection-planning terms:**

“botulinum toxin” OR onabotulinumtoxinA OR incobotulinumtoxinA OR abobotulinumtoxinA OR BoNT-A OR “injection technique” OR “injection point” OR “treatment planning”

Searches combined the three blocks with the AND operator. Backward and forward citation tracking of key papers complemented the database search to capture cadaveric, EMG, and clinical references not retrieved by the database query.

**S3. Inclusion and exclusion criteria**

**Inclusion criteria.**

Studies were considered eligible when they reported human, muscle-level mapping data relevant to upper-face injection planning. Eligible study designs included cadaveric and histological investigations, immunohistochemistry studies, modified Sihler-staining studies, three-dimensional cadaveric reconstructions, in vivo HD-sEMG studies, ultrasonographic anatomical descriptions, and clinical investigations that explicitly addressed injection point selection or muscle-specific targeting in the upper face.

**Exclusion criteria.**

Animal-only studies, studies of the lower face or non-mimetic musculature, studies whose primary focus was unrelated to NMJ topography or to upper-face BoNT-A planning, and conference abstracts without retrievable full text were not included. No filter was applied for sample size, given that several foundational anatomical contributions in this field rely on small cadaveric series.

**Language.**

No language restrictions were applied. English-language full texts were preferred when available; for non-English studies that fulfilled the eligibility criteria, translations were sourced as needed to ensure accurate extraction. Several included studies were performed in non-aesthetic clinical contexts (e.g., disease populations such as blepharospasm); this is acknowledged as a contextual limitation in §4.6 of the main text.

**S4. Screening and synthesis procedure**

Records were exported to a spreadsheet and deduplicated. Title/abstract screening was followed by full-text assessment; both stages were performed by a single reviewer. Single-reviewer screening, extraction, and qualitative integration constitute a known source of selection and interpretation bias and limit the reproducibility of the synthesis; this is also stated in the Limitations section of the main text.

Evidence was synthesized qualitatively by muscle, emphasizing NMJ cluster location, depth, and distribution patterns. Given heterogeneous methods and inter-individual variability, translational implications are presented as anatomy-informed guidance rather than prescriptive injection rules.

**S5. Construction of Figure 1 (depth-aware probabilistic map)**

In integrating the available data for Figure 1, evidence was prioritized in the following hierarchical order: (i) direct human histological and immunohistochemical visualization of NMJs; (ii) in vivo HD-sEMG, as a functional proxy of endplate activity; (iii) macroscopic intramuscular nerve arborization, as an anatomical proxy of likely endplate territories; and (iv) cadaveric topographic measurements, used to anchor surface coordinates and approximate depth. No formal consensus method (Delphi, nominal group technique, or similar) was applied. The source-by-source contributions to the integrated map are detailed in Supplementary Table 1.
