# Supplementary material for: The Neuromuscular Junction Distribution in the Upper Face: An Anatomy‐to‐Practice Review to Inform Botulinum Toxin Type A Treatment Planning
Source: J Cosmet Dermatol. 2026 May 13;25:e70921. doi: 10.1111/jocd.70921 (PMC13172661; doi:10.1111/jocd.70921)
Supplement: Supplementary file 2 — Table S1: Anatomical evidence underlying the depth‐aware probabilistic map of upper‐face motor zones (Figure 1). [file JOCD-25-e70921-s002.docx]

**Suplementary Table.** **Anatomical evidence underlying the depth-aware probabilistic map of upper-face motor zones (Figure 1).**

| **Muscle** | **Study (Reference no.)** | **Evidence type** | **Localization modality** | **Principal NMJ-relevant finding** |
| --- | --- | --- | --- | --- |
| **Frontalis** | Welter, Bramke, May (2022)⁸ | Direct human histology / immunohistochemistry | Cadaveric mapping | NMJs aggregate centrally within the muscle belly, tethered to the deep fascial aspect; dense atypical nerve endings at the superior transition zone with the galea aponeurotica. |
|  | Han et al. (2025)¹⁵ | Human anatomical mapping | Distal nerve endings as topographic proxies | Higher density of distal nerve endings toward the middle and upper thirds of the frontalis belly; sparse superior and inferior peripheral regions. |
|  | Neubert (2016)¹⁶ | In vivo HD-sEMG | Functional endplate localization | Endplates predominantly localized in the upper half of the forehead; organized either as a continuous transverse band or as two clusters (medial and lateral). |
|  | Yi et al. (2024)¹⁷ | Cadaveric anatomy | Surface landmarks + thickness measurements | Surface-anchored injection points based on external landmarks of the forehead and on regional variation in frontalis thickness (anatomical localization guidance, not direct NMJ mapping). |
| **Corrugator supercilii** | Janis et al. (2007)²¹ | Cadaveric topography | Macroscopic measurements | Topographic anchoring of muscle origin and trajectory used to support surface coordinates of the medial CSM motor zone. |
|  | Lee et al. (2020)²² | 3D cadaveric mapping | Three-dimensional reconstruction | Depth and territory; basis for the consolidated 5.4–6.6 mm depth estimate of the CSM motor zone from the skin surface. |
|  | Hwang, Lee, Lim (2017)¹⁸ | Systematic review of anatomy | Anatomical synthesis | Depth of the CSM surface remains relatively constant along its lateral course. |
|  | Goodmurphy & Ovalle (1999)²⁰ | Histological evaluation (biopsy) | Microscopic visualization | Confirmed presence of microscopic NMJ clusters within the corrugator supercilii muscle. |
|  | Neubert (2016)¹⁶ | In vivo HD-sEMG | Functional endplate localization | Endplates absent in the lateral third of the CSM; concentrate as a functional cluster in the medial third. |
| **Orbicularis oculi** | Wirtschafter et al. (1994)²⁴ | Histological mapping | Cadaveric | Pretarsal NMJs diffusely scattered across the entire eyelid; preseptal NMJs concentrate medially and laterally, sparse centrally. |
|  | Çiçek et al. (2024)¹⁰ | Modified Sihler stain | Cadaveric (clock model) | Higher nerve-branch density superolaterally above the palpebral fissure and below it (right: 4–7 o’clock; left: 5–8 o’clock). |
|  | Happak et al. (1997)⁹ | Human histology | Direct visualization | NMJs evenly spread across the muscle, isolated or grouped into many small motor zones; multifocal innervation common, with individual fibers bearing 2–5 NMJs. |
|  | Borodic et al. (1991)¹ | Histological observation | Direct visualization | Diffuse innervation pattern of the orbicularis oculi consistent with multifocal NMJ distribution. |
|  | Neubert (2016)¹⁶ + Barth (2017)²⁵ | In vivo HD-sEMG | Functional endplate localization | Endplates over the entire muscle with high inter-individual variability; clusters lateral to outer canthus, latero-cranial part, and cranial and caudal to the pupil. |
| **Depressor supercilii** | Cook, Lucarelli, Lemke (2001)²⁶ | Cadaveric anatomy | Macroscopic | Anatomically and histologically distinct muscle; origin ~10 mm above the medial canthal tendon; insertion ~14–15 mm superior to the MCT. |
|  | Neubert (2016)¹⁶ | In vivo HD-sEMG | Functional endplate localization | NMJs clustered in the central–caudal portion of the muscle, lateral glabella, along the orbital rim, superior to the medial canthus. |
| **Procerus** | Lorenc et al. (2013)² | Topographic measurement | Cadaveric | Procerus belly located at 2–3 mm beneath the skin; thickness <1 mm. |
|  | Nemoto (2001)²³ + Caminer et al. (2006)²⁸ + Hwang, Kim, Chung (2004)²⁹ | Macroscopic dissection | Cadaveric | Motor nerve typically penetrates the procerus at its temporal margin and posterior surface. |
|  | Neubert (2016)¹⁶ | In vivo HD-sEMG | Functional endplate localization | NMJ clusters in the inferior portion of the muscle near its origin; loosely distributed mediolaterally; rarely identified in superior positions. |

**Hierarchy of evidence used to weight contributions in Figure 1:** direct human histology and immunohistochemistry > in vivo high-density surface electromyography (HD-sEMG) > macroscopic intramuscular nerve arborization > cadaveric topographic measurements (used to anchor surface coordinates and approximate depth). No formal consensus method (Delphi or similar) was applied; Figure 1 represents a single-author qualitative integration of the published anatomical evidence, intended as an anatomical reference rather than as a prescriptive injection template.

**Abbreviations:** NMJ, neuromuscular junction; CSM, corrugator supercilii muscle; MCT, medial canthal tendon; HD-sEMG, high-density surface electromyography.
